# Low-Dose Endothelial-Monocyte-Activating Polypeptide-II Induced Autophagy by Down-Regulating miR-20a in U-87 and U-251 Glioma Cells

**DOI:** 10.3389/fncel.2016.00128

**Published:** 2016-05-17

**Authors:** Jiajia Chen, Libo Liu, Yunhui Liu, Xiaobai Liu, Chengbin Qu, Fanjie Meng, Jun Ma, Yang Lin, Yixue Xue

**Affiliations:** ^1^Department of Neurobiology, College of Basic Medicine, China Medical UniversityShenyang, China; ^2^Institute of Pathology and Pathophysiology, China Medical UniversityShenyang, China; ^3^Department of Neurosurgery, Shengjing Hospital of China Medical UniversityShenyang, China; ^4^Liaoning Research Center for Translational Medicine in Nervous System DiseaseShenyang, China

**Keywords:** EMAP-II, U-87, U-251, miR-20a, autophagy, LC3, ATG7, ATG5

## Abstract

Preliminary studies have shown that endothelial-monocyte-activating polypeptide-II (EMAP-II) induces autophagy and inhibits the viability of glioma cells via an unknown molecular mechanism. This study explored the possible mechanisms associated with EMAP-II-induced autophagy in glioma cells by regulation of the expression of microRNA-20a (miR-20a). EMAP-II effectively inhibited the viability, migration and invasion of human U-87 and U-251 glioma cells. EMAP-II also up-regulated the expression level of autophagy biomarker microtubule-associated protein one light chain 3 (LC3)-II/I, autophagy related gene ATG7 and ATG5, but down-regulated autophagy substrate P62/SQSTM1 protein expression. The expression levels of miR-20a decreased significantly after U-87 and U-251 cells were treated with EMAP-II. MiR-20a overexpression partly reversed the EMAP-II-induced up-regulation of LC3-II/I and down-regulation of P62/SQSTM1. MiR-20a had a negative regulatory effect on the expression of the proteins ATG7 and ATG5; which were also targets of miR-20a, as detected by a dual-luciferase reporter assay. In addition, both EMAP-II and miR-20a inhibition significantly reduced the viability, migration and invasion of U-87 and U-251 cells, and their combination showed a synergistic effect. Furthermore, nude mice carrying silencing-expressed miR-20a combined with EMAP-II treatment produced the smallest tumors and the highest survival. In summary, low-dose EMAP-II increased expression levels of ATG5 and ATG7 via down-regulation of the expression of miR-20a. This activated the autophagy pathway, thereby significantly inhibiting the viability, migration and invasion of U-87 and U-251 glioma cells. The combined treatment of EMAP-II with a miR-20a inhibitor showed a synergistic effect against glioma.

## Introduction

Glioma is regarded as the most prevalent primary malignant intracranial neoplasm. Glioblastoma is the type of glioma that accounts for 50–60% of malignant brain tumors (Jakab et al., 2011). Glioblastoma has been defined as a grade III-IV malignant tumor by the World Health Organization due to its high degree of malignancy and active mitosis. Existing treatments for glioblastoma include surgery, postoperative radiotherapy, chemotherapy, and other adjuvant therapies; however, treatment efficacy is poor due to its high malignancy, strong invasiveness, and resistance to radiotherapy and chemotherapy (McLendon and Halperin, 2003; Wen and Kesari, 2008; Bartek et al., 2012). Therefore, a novel strategy is urgently needed for glioblastoma treatment.

Endothelial-monocyte-activating polypeptide-II (EMAP-II), a multifunctional polypeptide, has pro-inflammatory and antiangiogenic activities (Mogylnytska, 2015). EMAP-II has been widely applied as a treatment because it inhibits the growth, proliferation and metastasis of primary and metastatic tumors (Schwarz et al., 1999). EMAP-II significantly inhibits the activity of pancreatic ductal adenocarcinoma cells (Schwarz et al., 2010) and exerts anti-cancer effects in prostate adenocarcinoma xenografts (Reznikov et al., 2007). Our preliminary studies have demonstrated that low-dose EMAP-II treatment can increase permeability of the blood-tumor barrier (BTB; Xie et al., 2010). These results indicate that the anti-tumor effects of EMAP-II are mediated by opening of the BTB, which enables penetration of anticancer drugs. Our previous findings showed that low-dose (0.05 nM) EMAP-II directly induces apoptosis of rat C6 glioma cells via the mitochondrial pathway (Liu et al., 2015b); EMAP-II induces autophagy of U-118 glioma cells in addition to inhibiting their activity (Liu et al., 2013). This demonstrates that EMAP-II suppresses the viability of glioma cells through apoptosis or autophagy, which leads to an anti-tumor effect.

Autophagy is an evolutionarily conserved process that is often activated under cellular stress (Kroemer et al., 2010). Autophagy can protect cells from damage, but can also induce cell death (Shen and Codogno, 2011). Studies have increasingly observed death of tumor cells due to autophagy (Akar et al., 2008; Seong et al., 2014). Temozolomide, a sensitive drug for treatment of glioma, causes G2/M phase arrest in glioma cells and induces autophagic cell death instead of apoptosis (Kanzawa et al., 2003, 2004). We speculate that malignant glioblastoma is more sensitive to autophagic cell death pathways (Lefranc et al., 2005, 2006) and that EMAP-II may induce glioma cell death through autophagy, but the molecular mechanism remains unclear.

MicroRNA is highly involved in the regulation of various physiological and pathological responses in cells (Bartel, 2004, 2009; Ebert and Sharp, 2012), and it can also modulate autophagic signaling networks, most notably in cancer (Frankel et al., 2011; Fu et al., 2012; Korkmaz et al., 2012). MicroRNA-20a (MiR-20a), a miR-17-92 family member located on chromosome 13, is highly expressed in glioma tissues; overexpression of miR-20a promotes proliferation of U-251 glioma cells, suggesting a tumor-promoting effect (Yao et al., 2012). Furthermore, miR-20a is also considered as an autophagy-related gene (ATG) and it may participate in regulating leucine deprivation-induced autophagy by changing intracellular levels of key autophagy-related proteins in C2C12 myoblasts (Wu et al., 2012). These findings suggest that low miR-20a expression contributes to up-regulation of autophagy-related protein expression, activates autophagy, and inhibits the proliferation of glioma cells. Whether EMAP-II induces autophagy through down-regulation of miR-20a expression, and thus affects the viability of glioma cells, is not known.

This study aims to verify whether low-dose (0.05 nM) EMAP-II treatment induces autophagy in U-87 and U-251 glioma cells by regulation of the expression of miR-20a, and explores the molecular mechanisms associated with EMAP-II-induced glioma cell autophagy.

## Materials and Methods

### Cell Lines and Cell Cultures

Human malignant glioma cell line U-87, U-251 and human embryonic kidney HEK293T cell line were purchased from Shanghai Institutes for Biological Sciences and Cell Resource Center (MD, USA). U-87 and HEK293T cells were cultured in DMEM supplemented with 10% fetal bovine serum (FBS, Life Technologies Corporation, Paisley, UK). U-251 cells were cultured in DMEM/F12 supplemented with 10% FBS. All cells were cultured at 37°C in an atmosphere of 5% CO_2_ and 95% air.

### Experimental Groups

To test the effect of EMAP-II on U-87 and U-251 cells, the experiments were divided into five groups (*n* = 8): (1) Control group, cells were treated with 0.9% sodium chloride (NS); (2) EMAP-II 0.5 h group, cells were treated with EMAP-II for 0.5 h; (3) EMAP-II 1 h group, cells were treated with EMAP-II for 1 h; (4) EMAP-II 3 h group, cells were treated with EMAP-II for 3 h; and (5) EMAP-II 6 h group, cells were treated with EMAP-II for 6 h. EMAP-II (Sigma–Aldrich, St. Louis, MO, USA) was dissolved in 0.9% sodium chloride, and 0.05 nM was selected as the optimal concentration for our investigation according to our previous research (Liu et al., 2013).

To investigate whether autophagy or apoptosis was involved in the process of EMAP-II regulating glioma cells, the autophagy inhibitor 3-Methyladenine (3-MA; Sigma–Aldrich, St. Louis, MO, USA) or apoptosis inhibitor Z-VAD-FMK (Z-VAD; Sigma–Aldrich, St. Louis, MO, USA) were administered before EMAP-II. 3-MA (2 mM) and Z-VAD (100 μm) were given 1 h prior to EMAP-II administration. Cells were divided into eight groups (*n* = 8): (1) Control group, cells were treated with 0.9% sodium chloride; (2) EMAP-II group, cells were treated with EMAP-II for 0.5 h; (3) 3-MA group, cells were treated with 3-MA for 1 h; (4) EMAP-II + 3-MA group; (5) Z-VAD group, cells were treated with Z-VAD for 1 h; (6) EMAP-II + Z-VAD group; (7) 3-MA + Z-VAD group; (8) EMAP-II + 3-MA + Z-VAD group.

In order to study the effect of miR-20a on EMAP-II inducing autophagy of U-87 and U-251 glioma cells, the experiments were divided into 10 groups (*n* = 8): (1) Control group; (2) EMAP–II group; (3) miR-20a (+) NC group, transfected with negative control of miR-20a overexpression; (4) miR-20a (+) group, transfected with miR-20a overexpression; (5) miR-20a (−) NC group, transfected with negative control of miR-20a silencing; (6) miR-20a (−) group, transfected with miR-20a silencing; (7) EMAP-II + miR-20a (+) NC group; (8) EMAP-II + miR-20a (+) group; (9) EMAP-II + miR-20a (−) NC group; and (10) EMAP-II + miR-20a (−) group.

To further verify the regulation effect of miR-20a on ATG7 and ATG5 expression, the experiments were divided into five groups: (1) Control group; (2) miR-20a (+) NC group; (3) miR-20a (+) group; (4) miR-20a (−) NC group; and (5) miR-20a (−) group.

To study the effect of EMAP-II and miR-20a inhibitor alone and in combination on cell proliferation, migration, and invasion, U-87 and U-251 cells were divided into six groups (*n* = 8): (1) Control group; (2) EMAP-II group; (3) miR-20a (−) NC group; (4) miR-20a (−) group; (5) NS + miR-20a (−) NC group, treatment with 0.9% sodium chloride after negative control of miR-20a silencing transfection; and (6) EMAP-II + miR-20a (−) group.

### Cell Transfection

Cells were seeded on six-well plates cultured overnight, then transfected with miR-20a mimic, miR-20a inhibitor, or their respective negative control (GenePharma, Shanghai, China) using lipofectamine 2000 reagent (Life Technologies Corporation) according to the manufacturer’s instructions. After 6 h transfection, the medium was removed and replaced with fresh medium. The cells were harvested after 48 h. After the cell clones of miR-20a overexpression or silencing were established, EMAP-II was administrated for 0.5 h.

### Cell Viability Assay

Cell viability was analyzed by MTT (Solarbio Company, Beijing, China) assay. Cells were seeded in 96-well plates at a density of 5 × 10^3^ cells/well. Cells were incubated overnight and then treated with EMAP-II (0.05 nM) for 0.5, 1, 3 and 6 h, while treatments with 0.9% sodium chloride as control. Then, the medium was replaced with fresh medium, and the cells were cultured at 37°C for 24 h. Afterward, 5 mg/mL MTT medium was added to each well and incubated for another 4 h. Then the media were carefully removed and the formazan crystals were solubilized by 150 μL DMSO (Sigma-Aldrich, St. Louis, MO, USA). Afterward, the optical density (OD) values were determined by spectrophotometry at 570 nm.

### Cell Migration and Invasion Assay

Cell migration and invasion abilities were evaluated by 24-well transwell chamber with 8 um pore size (Costar, USA). Cells were suspended in 100 μL serum-free medium and then added to the upper chamber (without Matrigel for cell migration assay) or seeded on the upper chambers pre-coated with Matrigel (for cell invasion assay), while 500 μL medium with 10% FBS was added to the lower chamber. EMAP-II (0.05 nM) was added to lower chamber for 0.5, 1, 3 and 6 h. Then, the medium in lower chamber was replaced with fresh medium. After incubation for 24 h, cells migrated or invaded to the bottom of the membrane were fixed and then stained with 20% Giemsa. Chambers were subjected to a microscopic inspection and counted.

### Western Blot Analysis

Protein concentration of U-87 and U-251 glioma cells was determined by the BCA protein assay (Beyotime Institute of Biotechnology, Jiangsu, China). Proteins (20–40 μg) were separated by 10–12% SDS-PAGE and transferred onto nitrocellulose membrane. The membranes were blocked by 5% non-fat milk for 2 h and then incubated overnight with primary antibodies against microtubule-associated protein one light chain 3 (LC3, 1:1000, Abcam, Cambridge, MA, USA, ab63817), ATG7(1:1000, Abgent, San Diego, CA, USA, AP1813c), ATG5 (1:1000, Proteintech, Chicago, IL, USA, 10181-2-AP), P62/SQSTM1 (1:1000, Abcam, Cambridge, MA, USA, ab56416), GAPDH (1:10,000, Proteintech, Chicago, IL, USA, 6000-4-Ig), respectively. After washing with TBST, The membranes were then incubated with secondary antibodies. The protein bands were visualized with enhanced chemiluminescence (ECL) kit (Santa Cruz Biotechnology) and scanned with Chemi Imager 5500 V2.03 Software.

### RNA Extraction and Quantitative RT-PCR

Total RNA was isolated using Trizol reagent (Life Technologies Corporation, Carlsbad, CA, USA) according to the manufacturer’s instructions. Real-Time PCR analysis was performed to test the expression levels of miR-20a, ATG7 and ATG5 by means of a 7500 Fast Real-Time PCR System. For quantification of miR-20a expression, reverse transcription and real-time PCR amplification were carried out using Taqman MicroRNA Reverse Transcription Kit and Taqman Universal Master Mix II with the TaqMan MicroRNA Assay of miR-20a and U6 (Applied Biosystems, Foster, CA, USA), respectively. For quantification of ATG7 and ATG5 mRNA, reverse transcription and real-time PCR amplification were carried out using RT-PCR kit and SYBR Premix Dimer Eraser (TaKaRa, Dalian, China) with the gene expression assays of ATG7, ATG5 and GAPDH (Applied Biosystems), respectively. Fold changes were calculated by relative quantification (2^−△△ Ct^) method.

### Immunofluorescence Staining

Cells were seeded onto cover slips in the six-well plates and incubated for 24 h. Cells were stained with LysoTracker Red at a final concentration of 50 nM. Then, cells were fixed in 4% paraformaldehyde for 20 min, blocked with 5% BSA for 2 h, and incubated with the primary antibody for LC3 (1:150, Abcam, Cambridge, MA, USA, ab63817) or P62/SQSTM1 (1:500, Abcam, Cambridge, MA, USA, ab56416) at 4°C overnight. After that, cells were washed and incubated with a secondary antibody conjugated to Alexa Fluor 488 and Alexa Fluor 555 (Beyotime Institute of Biotechnology, Jiangsu, China). The cells were then counterstained with DAPI (Beyotime Institute of Biotechnology, Jiangsu, China) for 10 min and visualized with a confocal microscope.

### Reporter Vectors Constructs and Luciferase Assays

Putative binding site between the 3′UTR of ATG7 (ATG5) mRNA and the seed region of miR-20a were predicted by TargetScan Human Release 6.2. HEK293 cells were seeded into a 96-well plate and cultured overnight at 37°C. After that, cells were co-transfected with the wild-type or mutated ATG7 (ATG5)-3′UTR reporter plasmid (GenePharma, Shanghai, China), and transfected with miR-20a mimic or miR-20a mimic NC. Luciferase assays were performed 48 h later using the Dual-Luciferase Reporter Assay System (Promega, Beijing, China).

#### Tumor Xenograft Implantation in Nude Mice

The stably transfected U-87 and U-251 cells were used in the *in vivo* study. Lentivirus encoding miR-20a silencing was generated using pLenti6.3/V5eDEST Gateway Vect—or Kit (Life Technologies Corporation, Carlsbad, CA, USA). The miR-20a silencing was ligated into the pLenti6.3/V5eDEST vector (GenePharma, Shanghai, China) and then pLenti6.3/V5eDEST-miR-20a-scilecing vector was generated. Lentivirus was generated in 293FT cells using the ViraPower Packaging Mix. After infection, the stable expressing cells of miR-20a-silencing [miR-20a (−)] were picked.

Four-week-old BALB/C athymic nude mice were purchased from the Vital River Company (Beijing, China). All experiments were carried out under the approval of the Administrative Panel on Laboratory Animal Care of China Medical University. The animals were free to autoclaved food and water during the study. For subcutaneous implantation, 3 × 10^5^ cells were inoculated into the right upper flank regions of the mice. Tumor volume was measured every 5 days when the tumors were apparently seen and calculated by the formula: volume (mm^3^) = length × width^2^/2. the mice were treated with 80 ng/kg EMAP-II or 0.9% sodium chloride every 12 h. For survival analysis in orthotopic inoculations, 3 × 10^5^ cells were stereotactically implanted into the right striatum of the mice. Mice were sacrificed by CO2 inhalation and death was confirmed by cervical dislocation if they exhibited excessive weight loss of 20% body weight, tumor metastasis, lethargy, or other signs of distress consisted with IACUC standards. The number of survived nude mice was recorded and survival analysis was performed using Kaplan Meier survival curve. When the tumors grew to about 80 mm^3^, the tumor-bearing mice were divided into four groups: (1) Control group, treated with 0.9% sodium chloride; (2) EMAP-II group, treated with 80 ng/kg EMAP-II; (3) miR-20a (−) group, treated with miR-20a (−) stable expressing cells; and (4) EMAP-II + miR-20a (−) group, treated with miR-20a (−) stable expressing cells and 80 ng/kg EMAP-II.

#### Statistical Analysis

Data were presented as the mean ± standard deviation (SD) and conducted with SPSS 13.0 (SPSS, IL, USA). Statistical analysis was performed using student’s *t*-test and the one-way ANOVA. *P* < 0.05 was considered to be statistically significant.

## Results

### Low-Dose EMAP-II Inhibited the Viability, Migration and Invasion Ability of U-87 and U-251 Cells

**Figure 1:**
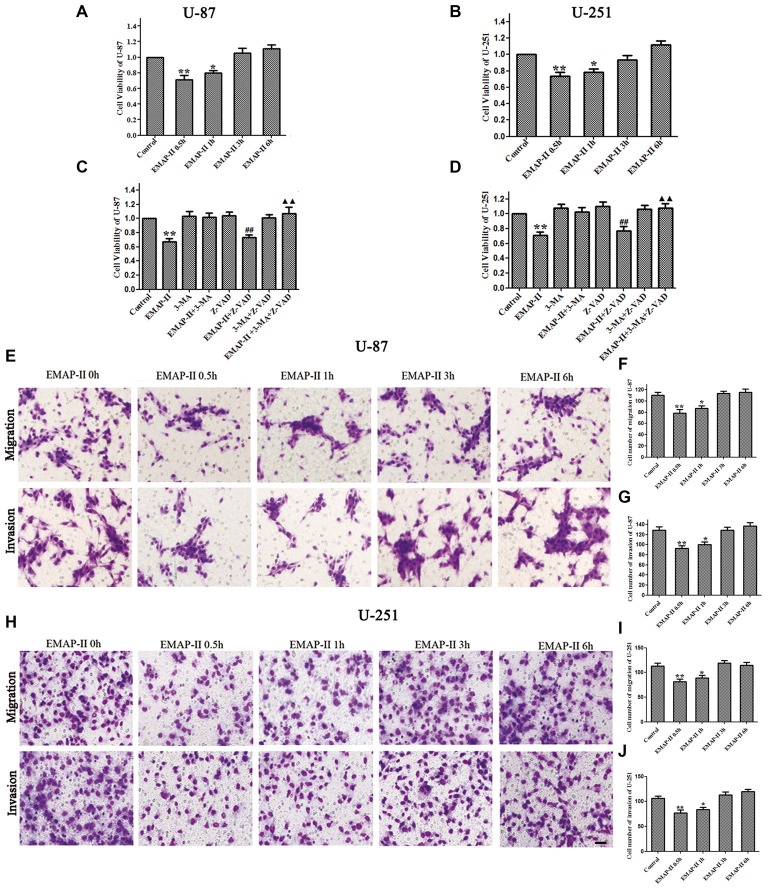
**Effects of endothelial-monocyte-activating polypeptide-II (EMAP-II) on proliferation, migration and invasion in U-87 and U-251 glioma cells. (A)** Effect of EMAP-II on growth inhibition in U-87 glioma cells. **(B)** Effect of EMAP-II on growth inhibition in U-251 glioma cells. **(C)**Effect of EMAP-II on growth inhibition in U-87glioma cells combined with 3-Methyladenine (3-MA) or Z-VAD. **(D)** Effect of EMAP-II on growth inhibition in U-251glioma cells combined with 3-MA or Z-VAD. **(E–G)** Quantification of cell migration and invasion of U-87 cells after treated with EMAP-II. **(H–J)** Quantification of cell migration and invasion of U-251 cells after treated with EMAP-II. Data is given as mean ± standard deviation (SD) of *n* = 8, *P < 0.05, **P < 0.01 vs. control group; ^##^*P* < 0.01 vs. Z-VAD group; ^▴▴^*P* < 0.01 vs. EMAP-II + Z-VAD group. Scale bars represent 40 μm.

The effects of EMAP-II on the viability, migration and invasion ability of U-87 and U-251 cells were evaluated by MTT and transwell assay, respectively. As shown in Figures 1A,B, the cell viability of U-87 and U-251 cells was inhibited by EMAP-II in a time-dependent manner. The inhibition effect on cell viability was most significant after cells were treated with EMAP-II for 0.5 h as compared to respective control group (*P* < 0.01). Meanwhile, compared with the control group, the migration and invasion ability of U-87 and U-251 cells were significantly decreased after EMAP -II treatment for 0.5 h and 1 h (*P* < 0.05, Figures 1E–J).

As shown in Figures 1C,D, there was no obvious difference among 3-MA, Z-VAD and 3-MA + Z-VAD groups compared with control group (*P* > 0.05), while EMAP-II significantly inhibited the cell viability (*P* < 0.01). 3-MA pretreatment significantly blocked EMAP-II’ effect on inhibiting the cell viability, and recovered the cell viability to the level in control group. The cell viability of EMAP-II + Z-VAD group was significantly decreased compared with Z-VAD group (*P* < 0.01), while there was no difference between EMAP-II + Z-VAD group and EMAP-II group (*P* > 0.05). These results suggested that Z-VAD had no impact on the inhibition effect of EMAP-II on the cell viability. There were no significant differences between EMAP-II + 3-MA + Z-VAD group and 3-MA + Z-VAD group or EMAP-II + 3-MA group. But the cell viability was significantly increased in EMAP-II + 3-MA + Z-VAD group compared with EMAP-II and EMAP-II + Z-VAD group. These evidences suggested that 3-MA could block the effect of EMAP-II on inhibiting the cell viability of U-87 and U-251 gioma cells, while Z-VAD had no such effect.

### The Effects of Low-Dose EMAP-II on the Protein Expression of LC3, P62/SQSTM1, ATG7 and ATG5 in U-87 and U-251 Cells

**Figure 2:**
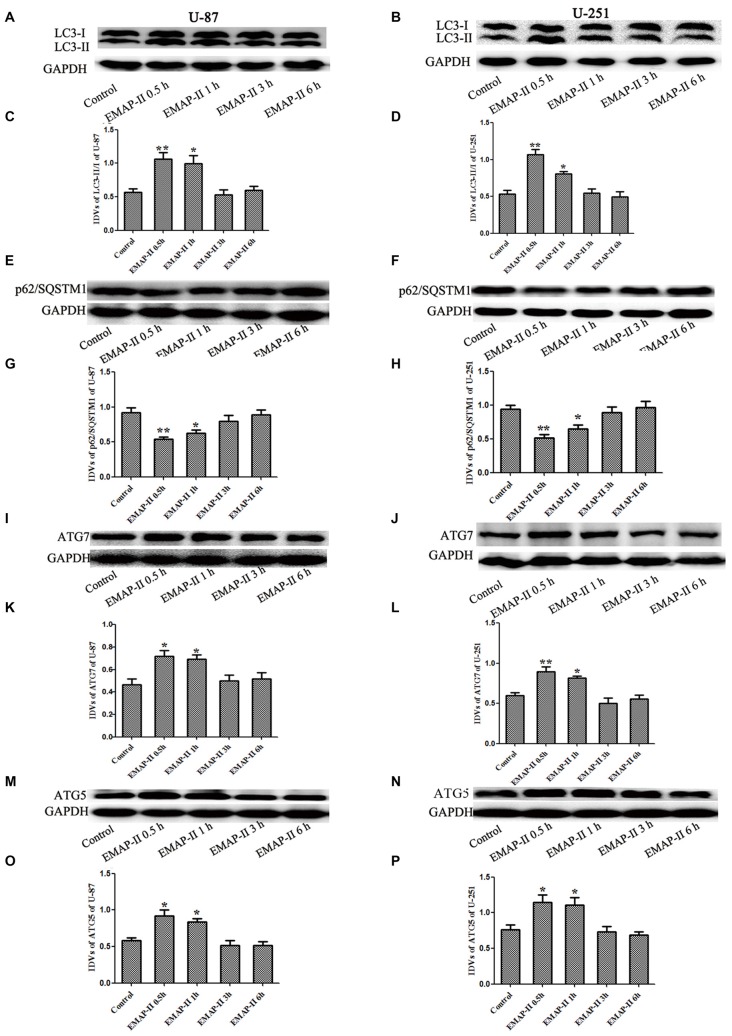
**Effects of EMAP-II on the expression of LC3, p62/SQSTM1, ATG7 and ATG5 in U-87 and U-251 glioma cells. (A,B,E,F,I,J,M,N)** Western blot analysis of the expressing levels of LC3, p62/SQSTM1, ATG7 and ATG5 in human U-87 glioma cells and U-251 glioma cells after treatment with EMAP-II for 0.5, 1, 3 and 6 h. **(C,D,G,H,K,L,O,P)** Western blot analysis of the LC3-II/-I, p62/SQSTM1/GAPDH, ATG7/GAPDH and ATG5/GAPDH levels in U-87 and U-251 glioma cells. Data is given as mean ± SD of *n* = 8. *P < 0.05, **P < 0.01 vs. control group.

Western blot assay was used to detect the protein expression of LC3, P62/SQSTM1, ATG7 and ATG5 of U-87 and U-251 cells after treatment with EMAP-II for 0.5, 1, 3 and 6 h. As shown in Figures 2A–D, the conversion of LC3-I to LC3-II in U-87 and U-251 cells increased at 0.5 h and 1 h compared with control group (*P* < 0.05), and peaked at 0.5 h (*P* < 0.01); However, there was no significant difference among 3 h, 6 h group and control group. The expression of P62/SQSTM1, an autophagy substrate, was significantly reduced in EMAP-II 0.5 h and 1 h groups compared with the control group (*P* < 0.05, Figures 2E–H) and the decline was most obvious at 0.5 h (*P* < 0.05). As shown in Figures 2I–P, the protein expression levels of ATG7 and ATG5 were significantly increased after EMAP-II treatment for 0.5 and 1 h, which was similar to LC3-II (*P* < 0.05). EMAP-II induced the autophagy of human U-87 and U-251 glioma cells, which might be correlated with the up-regulation of ATG7 and ATG5.

### The Effects of Low-Dose EMAP-II on the miR-20a Expression Levels in U-87 and U-251 Cells

**Figure 3:**
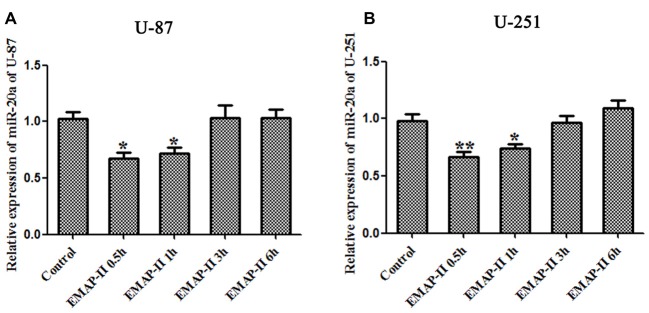
**Effects of EMAP-II on the expression of microRNA-20a (miR-20a) in U-87 and U-251 glioma Cells. (A,B)** The relative expression of miR-20a was detected by real-time quantitative PCR in U-87 and U-251 glioma cells after treatment with EMAP-II for 0.5, 1, 3 and 6 h. Data is given as mean ± SD of *n* = 8. *P < 0.05, **P < 0.01 vs. control group.

The expression levels of miR-20a in U-87 and U-251 cells after treating with EMAP-II were detected by quantitative real-time PCR. As shown in Figures 3A,B, compared with control group, EMAP-II significantly reduced the expression levels of miR-20a normalized to U6 at 0.5 h and 1 h and the decline was most obvious at 0.5 h (*P* < 0.05); meanwhile, there was no significant difference among EMAP-II 3 h, 6 h groups and control group (*P* > 0.05).

### The Effects of miR-20a on the Protein Expression of LC3 and P62/SQSTM1 in U-87 and U-251 Cells

**Figure 4:**
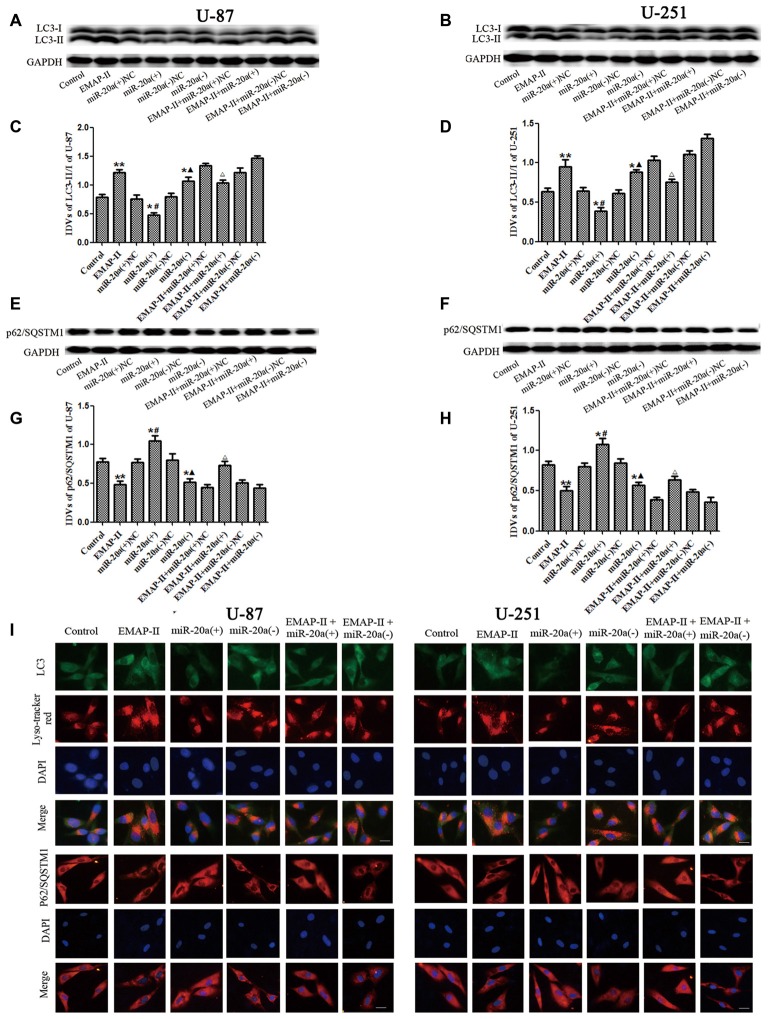
**The effects of miR-20a on protein expression levels of LC3 and P62/SQSTM1 in EMAP-II treated U-87 and U-251 glioma cells. (A,B,E,F)** Western blot analysis of the LC3-II/I and p62/SQSTM1 levels in human U-87 and U-251 glioma cells. **(C,D,G,H)** Western blot analysis of the LC3-II/I and P62/SQSTM1/GAPDH levels in U-87 and U-251 glioma cells. **(I)**The distribution and expression of LC3 and P62/SQSTM1 in U-87 and U-251 glioma cells was analyzed by immunofluorescence assay. Values are means ± SD (*n* = 8). *P < 0.05, **P < 0.01 vs. control group; ^#^*P* < 0.05 vs. miR-20a (+) NC group; ^▴^*P* < 0.05 vs. miR-20a (−) NC group; ^Δ^*P* < 0.05 vs. EMAP-II + miR-20a (+) NC group. Scale bar = 20 μm.

To further confirm whether miR-20a is involved in EMAP-II-induced autophagy, western blot and immunofluorescence assays were used to test the protein expression of LC3 and P62/SQSTM1 in U-87 and U-251 cells, which were transfected with a mimic or inhibitor of miR-20a. As shown in Figures 4A–H, the protein expression level of LC3-II/I significantly down-regulated and the protein expression level of P62/SQSTM1 significantly up-regulated in miR-20a (+) group compared with control and miR-20a (+) NC groups (*P* < 0.05). Whereas, miR-20a (−) group showed the opposite effect. The protein expression level of LC3-II/I significantly up-regulated and the protein expression level of P62/SQSTM1 significantly down-regulated in miR-20a (−) group when compared to control and miR-20a (−) NC groups. Compared with control group, EMAP-II significantly up-regulated LC3-II/I protein expression and down-regulated P62/SQSTM1 protein expression (*P* < 0.05). There was no significant difference among EMAP-II, EMAP-II + miR-20a (+) NC and EMAP-II + miR-20a (−) NC groups. Compared with EMAP-II + miR-20a (+) NC group, EMAP-II + miR-20a (+) significantly down-regulated LC3-II/I protein expression and up-regulated P62/SQSTM1 protein expression (*P* < 0.05), whereas EMAP-II + miR-20a (−) group showed no significant differences.

Immunofluorescence staining was used to detect the distribution and expression of LC3 and P62/SQSTM1 in U-87 and U-251 cells. As shown in Figure 4I, the LC3 expression significantly increased in EMAP-II, miR-20a (−) and EMAP-II + miR-20a (−) groups, while decreased in miR-20a (+) group and there was no significant change in EMAP-II + miR-20a (+) group when compared to respective control group. Compared with EMAP-II group, the expression of LC3 decreased in EMAP-II + miR-20a (+) group and there was no significant difference in EMAP-II + miR-20a (−) group. In addition, there was a significant overlap between LC3 and lysosomal signals. The expression change of P62/SQSTM1 was reversed to LC3, which was decreased in EMAP-II, miR-20a (−) and EMAP-II + miR-20a (−) groups, while increased in miR-20a (+) group and there was no significant difference between EMAP-II + miR-20a (+) group and respective control groups. Compared with EMAP-II group, the expression of P62/SQSTM1 increased in EMAP-II + miR-20a (+) group and there was no significant difference in EMAP-II + miR-20a (−) group. These results indicated that miR-20a overexpression reversed the effect of EMAP-II up-regulating the expression of LC3-II/I and down-regulating the expression of p62/SQSTM1, and miR-20a might be involved in the regulating of EMAP-II inducing autophagy.

### MiR-20a Negatively Regulated ATG7 and ATG5 Protein Expression Levels in U-87 and U-251 Cells

**Figure 5:**
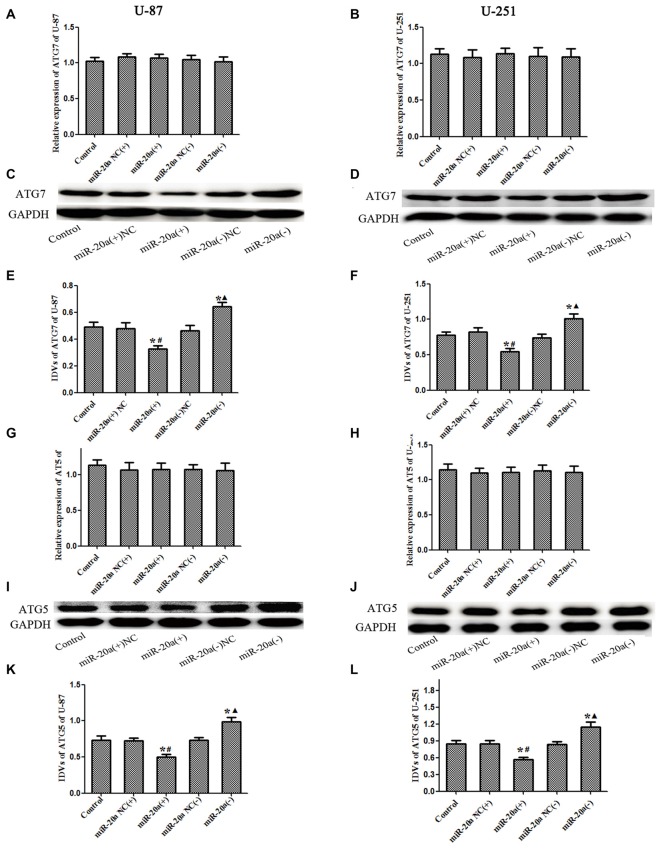
**ATG7 and ATG5 was changed after overexpression or silencing of miR-20ain U-87 and U-251 glioma Cells. (A,B,G,H)** The relative expression of ATG7 mRNA and ATG5 mRNA levels were detected by real-time quantitative PCR in U-87 and U-251 glioma cells after overexpression or silencing of miR-20a. **(C,D,I,J)** Western blot analysis of the ATG7 and ATG5 levels in human U-87 and U-251 glioma cells after overexpression or silencing of miR-20a. **(E,F,K,L)** Western blot analysis of the ATG7/GAPDH and ATG5/GAPDH levels in U-87 and U-251 glioma cells. Data is given as mean ± SD of *n* = 8. *P < 0.05 vs. control group; ^#^*P* < 0.05 vs. miR-20a (+) NC group; ^▴^*P* < 0.05 vs. miR-20a (−) NC group.

U-87 and U-251 cells were transfected with miR-20a mimics and its inhibitors, and then the mRNA and protein levels of ATG7 and ATG5 in U-87 and U-251 cells were detected by using western blot and quantitative real-time PCR, respectively. As shown in Figures 5A,B,G,H, there were no obvious differences of ATG7 mRNA and ATG5 mRNA levels between miR-20a (+) and miR-20a (+) NC groups, miR-20a (−) and miR-20a (−) NC groups (*P* > 0.05). As shown in Figures 5C–F, 5C–F,I–L, there were no obvious differences among control, miR-20a (+) NC and miR-20a (−) NC groups. The protein expression levels of ATG7 and ATG5 in miR-20a (+) group were significantly decreased compared with those of control group and miR-20a (+) NC group (*P* < 0.05). However, protein expression levels of ATG7 and ATG5 in miR-20a (−) group increased in comparison with those of control group and miR-20a (−) NC group (*P* < 0.05).

### MiR-20a Directly Targeted the 3′-UTR of ATG7 and ATG5

**Figure 6:**
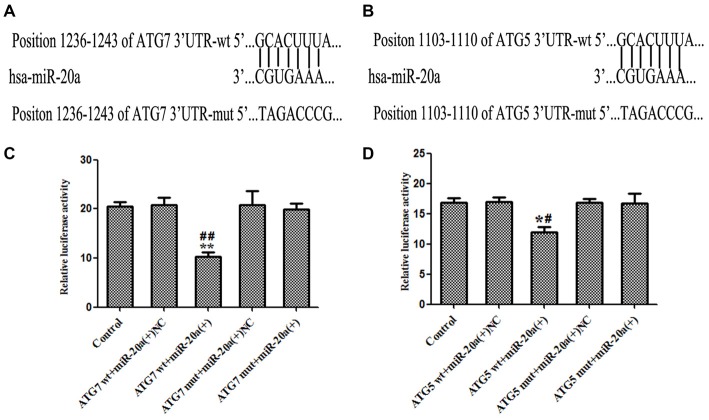
**ATG7 and ATG5 are the direct targets of miR-20a. (A,B)** The putative binding sites of ATG7 3′UTR and ATG5 3′UTR matching with the seed region of miR-20a were predicted with the help of TargetScan. **(C,D)** Relative luciferase activity was expressed as firefly/renilla luciferase activity. Values are means ± SD (*n* = 4, each). *P < 0.05, **P < 0.01 vs. control group; ^#^*P* < 0.05 vs. ATG5wt + miR-20a (+) NC group, ^##^*P* < 0.01 vs. ATG7 wt + miR-20a (+) group.

As shown in Figures 6A,B, by using TargetScan Human Release 6.2 Software^1^, 3′-UTR of ATG7 and ATG5 was predicted as a putative binding site matching with the seed region of miR-20a. To verify that ATG7 or ATG5 3′-UTR is a direct target of miR-20a, we cloned the reporter plasmid containing the wild 3′-UTR of ATG7 and ATG5 (atg7/ATG5-3UTR-Wt) and mutant 3′-UTR of ATG7 and ATG5 (atg7/ATG5-3UTR-mut). As shown in Figure 6C, compared with ATG7 wt + miR-20a (+) NC group, luciferase activity significantly decreased in ATG7wt + miR-20a (+) group (*P* < 0.01), while ATG7 mut + miR-20a (+) group showed no significant difference of luciferase activities with ATG7 mut + miR-20a (+) NC group (*P* > 0.05). Similarly, luciferase activity significantly decreased in ATG5 wt + miR-20a (+) group compared with ATG5 wt + miR-20a (+) NC group (*P* < 0.05), while ATG5 mut + miR-20a (+) group showed no significant differences of luciferase activities as compared to ATG5 mut + miR-20a (+) NC group (*P* > 0.05, Figure 6D). These results showed that miR-20a directly targeted the 3′-UTR of ATG7 and ATG5.

### The Single or Combined Application of EMAP-II and miR-20a Inhibitors on the Viability, Migration and Invasion of U-87 and U-251 Cells

**Figure 7:**
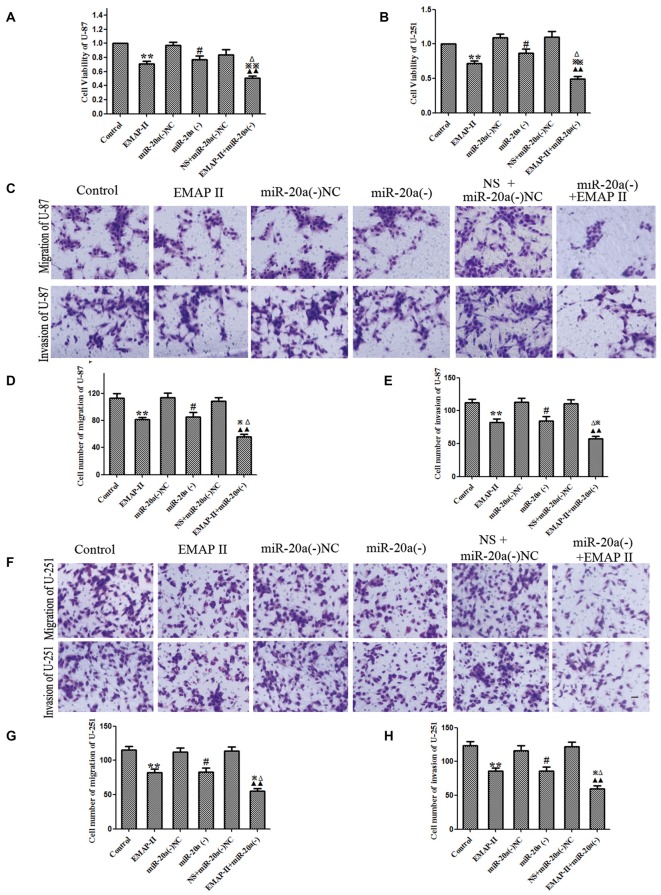
**EMAP-II alone and EMAP-II combined with miR-20a inhibitor inhibited the proliferation, migration and invasion of U-87 and U-251 Cells. (A,B)** The effects of EMAP-II and miR-20a inhibitor on growth inhibition and the migration of U-87 and U-251 cells were assessed by MTT. **(C–E)** Quantification of cell migration and invasion of U-87 cells. **(F–H)** Quantification of cell migration and invasion of U-251 cells. Data is given as mean ± SD of *n* = 8. *P < 0.05, **P < 0.01 vs. control group; ^#^*P* < 0.05 vs. miR-20a (−) NC; ^▴▴^*P* < 0.01 vs. NS + miR-20a (−) NC group; ^Δ^*P* < 0.05 vs. EMAP-II group; ^⋇^*P* < 0.05, ^⋇⋇^*P* < 0.01 vs. miR-20a (−) group. Scale bars represent 40 μm.

The functional role of EMAP-II and miR-20a inhibitor alone or in combination on cell viability, migration and invasion of U-87 and U-251 cells was determined. As shown in Figures 7A,B, compared with control group, the cell viability was decreased in EMAP-II, miR-20a (−) and EMAP-II + miR-20a (−) groups (*P* < 0.05). The combination of EMAP-II and miR-20a (−) displayed greater inhibitory effect than either EMAP-II or miR-20a (−) alone (*P* < 0.05). Similar to the above results, EMAP-II and miR-20a (−) groups inhibited the migration and invasion of cells, and the combination of EMAP-II and miR-20a (−) group displayed maximum inhibitory effect on the migration and invasion of cells (*P* < 0.05, Figures 7C–H).

### EMAP-II and miR-20a Inhibitor Inhibited Tumor Growth *In Vivo*

**Figure 8:**
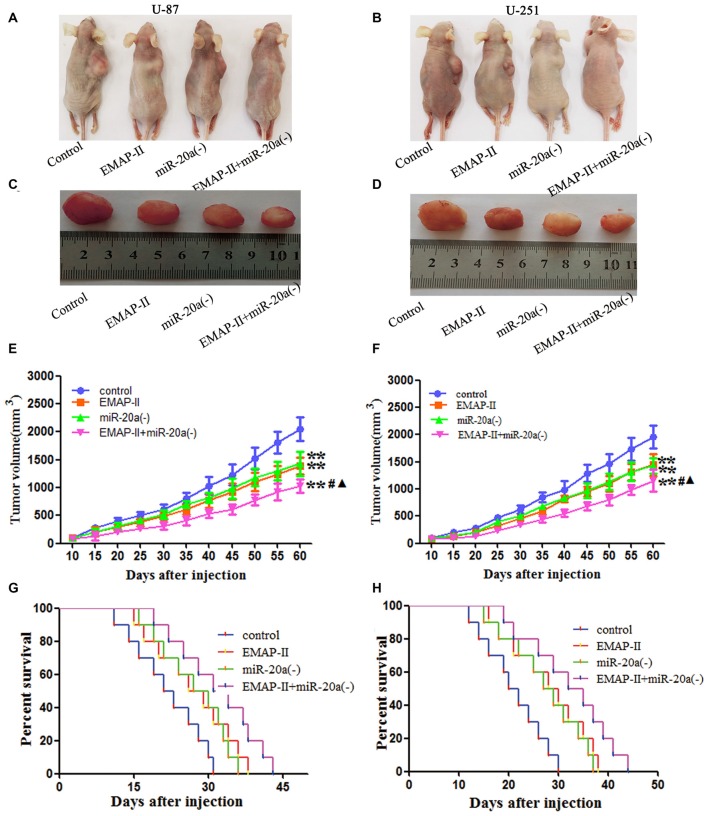
**EMAP-II and miR-20a inhibitor inhibited tumor growth *in vivo*. (A–D)** Represent active images of mice and tumors removed from the xenografted mice. **(E,F)** Tumor growth curves in nude mice. Tumor volume was calculated every 5 days after injection (*n* = 5). **(G,H)** The survival curves of nude mice injected (*n* = 10). **P < 0.01 vs. control group; ^#^*P* < 0.05 vs. EMAP-II group; ^▴^*P* < 0.05 vs. miR-20a (−) group.

The growth-inhibitory effect of EMAP-II and miR-20a inhibitor on U-87 and U-251 cells was further tested in xenografted mice. As shown in Figures 8A–F, tumor volumes of xenografts were significantly suppressed in EMAP-II, miR-20a (−) and EMAP-II + miR-20a (−) groups as compared to control group (*P* < 0.01), and the tumor volumes was most significantly decreased in EMAP-II + miR-20a (−) group (*P* < 0.01). Survival curve analysis showed that, survival time of nude mice was longer in EMAP-II, miR-20a (−) and EMAP-II + miR-20a (−) groups than that in control group, and was significantly longest in EMAP-II + miR-20a (−) group (Figures 8G,H). These data showed that nude mice were treated by EMAP-II combined with miR-20a inhibitor produced the smallest tumors and had the highest survival rate.

## Discussion

The present study showed that low doses of EMAP-II (0.05 nM) effectively inhibited the viability, migration, and invasion of human U-87 and U-251 glioma cells. This was reversed by the autophagy inhibitor 3-MA, while the apoptosis inhibitor Z-VAD had no effect. EMAP-II up-regulated the expression of the proteins LC3-II/I, ATG7 and ATG5, but down-regulated P62/SQSTM1 expression in the U-87 and U-251 glioma cells, demonstrating that the antitumor activity results from the induction of autophagy, not apoptosis. The expression levels of miR-20a decreased significantly after EMAP-II treatment. The EMAP-II-induced up-regulation of LC3-II/I, and down-regulation of P62/SQSTM1, was blocked by miR-20a overexpression; while miR-20a had a negative effect on ATG7 and ATG5 protein expression. The dual-luciferase reporter assay revealed that miR-20a binds to the 3′UTR of ATG7 and ATG5 mRNA. Results above demonstrated that EMAP-II up-regulates expression of ATG7 and ATG5 through miR-20a inhibition. This induced autophagy of the glioma cells. EMAP-II and miR-20a inhibition significantly reduced the viability, migration and invasion of U-87 and U-251 cells in a synergistic manner. Furthermore, nude mice carrying silencing-expressed miR-20a combined with EMAP-II treatment had the smallest tumors and highest survival.

EMAP-II is a potential anti-tumor substance with dual effects: opening the BTB and inhibiting tumor growth. This group has investigated the molecular mechanism of opening the BTB, but the anti-tumor effect is poorly understood. As a pro-inflammatory cytokine, EMAP-II triggers apoptosis of endothelial cells and inhibits tumor angiogenesis (Berger et al., 2000). EMAP-II decreases the viability of pancreatic ductal adenocarcinoma cells through inhibition of fibronectin-dependent proliferation (Schwarz et al., 2010); low-dose (0.05 nM) EMAP-II also induces the apoptosis of C6 glioma cells via the mitochondrial pathway (Liu et al., 2015b). In addition, EMAP-II activates the autophagy pathway and decreases the viability of U-118 glioma cells (Liu et al., 2013). Thus, EMAP-II can exert anti-tumor effects through a variety of pathways. This study found that low doses of EMAP-II significantly inhibit the viability, migration and invasion of U-87 and U-251 glioma cells by inhibiting biological activity.

Increasing evidence shows that programmed cell death is important for inducing tumor cell death, primarily by apoptosis or autophagy. Drug-induced autophagic cell death is an effective cancer treatment. For example, fluorouracil up-regulated expression of the protein Beclin-1 triggers autophagic cell death of gastric cancer cells (Yang and Pan, 2015). Dihydroartemisinin increased Beclin-1 and LC3-B expression levels, induced autophagy in glioma cells, inhibited cell viability, and enhanced the anti-tumor effect of temozolomide (Zhang et al., 2015). The autophagy inhibitor 3-MA and caspase inhibitor Z-VAD were applied to determine whether low-dose EMAP-II inhibits the biological activity of human U-87 and U-251 glioma cells through autophagy or apoptosis. Results showed that 3-MA can block EMAP-II-induced inhibition of U-87 and U-251 cell viability, while Z-VAD had no impact. The above findings indicated that low-dose EMAP-II reduced viability of tumor cells through autophagy, and was not associated with apoptosis. A previous study has reported that low doses of EMAP-II inhibited the viability of rat C6 glioma cells via apoptosis, in contrast to these findings showing the autophagy-based contribution of low-dose EMAP-II on U-87 and U-251 cells, but no evidence of apoptosis. The discrepancy could be explained by the different cell sources and strains that were used.

LC3 is a protein involved in the formation of the autophagosome. Synthetic LC3 is cut by ATG4 to make LC3-I, which binds the autophagic membrane with the help of ATG3 and ATG7, when it then transforms into active LC3-II. Therefore, the conversion ratio of LC3-I to LC3-II represents autophagy activity (Tanida et al., 2004). The results of this study found that EMAP-II significantly increased LC3-II/I expression in U-87 and U-251 glioma cells with a peak at 0.5 h, indicating that EMAP-II can induce cell autophagy. P62/SQSTM1 is degraded during autophagy, reflecting autolysosomal activity and autophagic flux (Ichimura and Komatsu, 2010). It was also shown that P62/SQSTM1, which is eliminated through autophagy, inhibits tumor growth (Moscat and Diaz-Meco, 2009). In this study, we found that EMAP-II significantly lowered P62/SQSTM1 expression, and the decline was most obvious at 0.5 h. This demonstrated that low-dose EMAP-II inhibits the viability of U-87 and U-251 glioma cells via autophagy.

To investigate the potential mechanism underlying EMAP-II-induced autophagy in U-87 and U-251 glioma cells, the effects of EMAP-II on the expression of ATG7 and ATG5 were studied. EMAP-II significantly increased ATG5 and ATG7 expression in U-87 and U-251 cells with a peak at 0.5 h. It is possible that EMAP-II induced autophagy of U-87 and U-251 cells by up-regulation of ATG7 and ATG5 expression. ATG7 serves as an E1-activating enzyme involved in autophagosome formation and vesicle progression (Weidberg et al., 2011). ATG7 knockout mice die within 1 day of birth and show reduced pup size, due to the impaired autophagy pathway (Tanzer and Stadler, 2004). ATG5 plays a crucial role in autophagosome elongation and apoptosis; even it is associated with the occurrence of tumors in various tissues (Liu et al., 2015a). These data indicate that EMAP-II induced autophagy of U-87 and U-251 cells by up-regulating ATG7 and ATG5 expression.

MiR-20a acts as a tumor promoter or suppressor, depending on the cell type. For example, miR-20a overexpression significantly inhibited the proliferation and invasive capacity of thyroid cancer cells (Xiong et al., 2014), while it is a cancer-promoter in cervical cells (Kang et al., 2012). MiR-20a expression significantly increased in glioma stem cells, which enhanced invasiveness (Wang et al., 2015); the degree of malignancy in brainstem gliomas was often higher in children than in adults, which correlated with up-regulation of miR-20a in pediatric brainstem gliomas (Wang et al., 2012). This indicates that miR-20a may serve as a cancer-promoting gene and affect the biological activity of glioma cells. Our study found that the expression level of miR-20a was significantly decreased in U-87 and U-251 glioma cells after EMAP-II treatment, suggesting EMAP-II inhibited the viability, migration, and invasion of glioma cells by decreasing miR-20a via an unknown mechanism.

It has been reported that miR-20a can affecte autophagy in C2C12 cells (Wu et al., 2012). However, whether EMAP-II induced autophagy of U-87 and U-251 cells via regulation of miR-20a is not clear. In this study, miR-20a overexpression prevented the EMAP-II-induced increase of LC3-II/I and decreases of P62/SQSTM1 expression. Research showed that miR-20a overexpression inhibits transformation from LC3-I to LC3-II, thereby negatively regulating autophagy in liver cancer cells (Chen et al., 2015). These results support the findings in this study. Therefore, EMAP-II can inhibit miR-20a expression and induce autophagy of U-87 and U-251 glioma cells by an unknown mechanism.

MiR-20a targets the ATG ULK1 post-transcriptionally and regulates autophagy of C2C12 cells (Wu et al., 2012), suggesting control by regulating expression of the autophagy-related protein. The results of this study showed that EMAP-II treatment of U-87 and U-251 cells led to a significant decrease in miR-20a expression and a significant increase in ATG7 and ATG5 expression. This suggests that EMAP-II up-regulation of ATG7 and ATG5 by down-regulation of miR-20a leads to autophagy. Using the bioinformatics Software TargetScan Human Release 6.2, putative binding sites for miR-20a were predicted on ATG7 and ATG5. The dual-luciferase reporter assay illustrated that miR-20a binds with the 3′-UTR of ATG7 and ATG5 mRNA, respectively. In addition, miR-20a overexpression significantly down-regulated ATG7 and ATG5 protein levels. However, neither miR-20a overexpression nor silencing affected mRNA expression. This suggests that miR-20a post-transcriptionally regulates ATG7 and ATG5. Therefore, EMAP-II may up-regulate expression of ATG5 and ATG7 via down-regulation of miR-20a, inducing autophagy in glioma cells.

The tumor-promoting effect of miR-20a has been reported in glioma cells and tissues (Yao et al., 2012). This study finds that EMAP-II and miR-20a inhibition have an antitumor effect toward glioma cells. Results showed that both EMAP-II and the miR-20a inhibitor effectively reduced cell viability, migration and invasion, and the combination brought synergistic or enhanced effects. A subsequent study analyzed the effect of EMAP-II and the miR-20a inhibitor on tumor size and survival time in a nude mouse model of subcutaneous xenograft. EMAP-II and the miR-20a inhibitor contributed to reduced tumor size and prolonged survival of the mice, highlighting the synergistic effect against glioma.

In summary, this is the first study proposing that low-dose EMAP-II treatment induces autophagy of U-87 and U-251 glioma cells leading to inhibition of cell viability, migration and invasion via the down-regulation of miR-20a. The mechanism of action is associated with negative regulation of the autophagy-related proteins ATG7 and ATG5 by miR-20a. Furthermore, combination of EMAP-II with a miR-20a inhibitor significantly reduces the proliferation, migration and invasion of glioma cells, prevents tumor growth *in vivo*, and exerts a synergistic effect against glioma. Therefore, EMAP-II combined with a miR-20a inhibitor shows promise as a new treatment for glioma.

## Author Contributions

Conceived and designed the experiments: YX, YL, and LL. Performed the experiments: JC and LL. Analyzed the data: XL, CQ, FM, and JM. Contributed reagents/materials/analysis tools: YL. Wrote the manuscript: JC, LL, and YX.

## Conflict of Interest Statement

The authors declare that the research was conducted in the absence of any commercial or financial relationships that could be construed as a potential conflict of interest.

## Footnotes

^1^http://www.targetscan.org
